# LSB-based pre-embedding video steganography with rotating & shifting poly-pattern block matrix

**DOI:** 10.7717/peerj-cs.843

**Published:** 2022-01-06

**Authors:** Murat Hacimurtazaoglu, Kemal Tutuncu

**Affiliations:** 1 Ardesen Vocational School, Computer Programming, Recep Tayyip Erdogan University, Rize, Turkey; 2 Electric Electronics Engineering, Selcuk University Technology Faculty, Konya, Turkey

**Keywords:** Data security, Information security, Steganography, Video steganography, Cryptography, Random number generation, Key block matrix

## Abstract

**Background:**

In terms of data-hiding areas, video steganography is more advantageous compared to other steganography techniques since it uses video as its cover medium. For any video steganography, the good trade-off among robustness, imperceptibility, and payload must be created and maintained. Even though it has the advantage of capacity, video steganography has the robustness problem especially regarding spatial domain is used to implement it. Transformation operations and statistical attacks can harm secret data. Thus, the ideal video steganography technique must provide high imperceptibility, high payload, and resistance towards visual, statistical and transformation-based steganalysis attacks.

**Methods:**

One of the most common spatial methods for hiding data within the cover medium is the Least Significant Bit (LSB) method. In this study, an LSB-based video steganography application that uses a poly-pattern key block matrix (KBM) as the key was proposed. The key is a 64 × 64 pixel block matrix that consists of 16 sub-pattern blocks with a pixel size of 16 × 16. To increase the security of the proposed approach, sub-patterns in the KBM are allowed to shift in four directions and rotate up to 270° depending on the user preference and logical operations. For additional security *XOR* and *AND* logical operations were used to determine whether to choose the next predetermined 64 × 64 pixel block or jump to another pixel block in the cover video frame to place a KBM to embed the secret data. The fact that the combination of variable KBM structure and logical operator for the secret data embedding distinguishes the proposed algorithm from previous video steganography studies conducted with LSB-based approaches.

**Results:**

Mean Squared Error (MSE), Structural Similarity Index (SSIM) and Peak Signal-to-Noise Ratio (PSNR) parameters were calculated for the detection of the imperceptibility (or the resistance against visual attacks ) of the proposed algorithm. The proposed algorithm obtained the best MSE, SSIM and PSNR parameter values based on the secret message length as 0.00066, 0.99999, 80.01458 dB for 42.8 Kb of secret message and 0.00173, 0.99999, 75.72723 dB for 109 Kb of secret message, respectively. These results are better than the results of classic LSB and the studies conducted with LSB-based video steganography approaches in the literature. Since the proposed system allows an equal amount of data embedding in each video frame the data loss will be less in transformation operations. The lost data can be easily obtained from the entire text with natural language processing. The variable structure of the KBM, logical operators and extra security preventions makes the proposed system be more secure and complex. This increases the unpredictability and resistance against statistical attacks. Thus, the proposed method provides high imperceptibility and resistance towards visual, statistical and transformation-based attacks while acceptable even high payload.

## Introduction

Most of the research study in video steganography is the extension of image steganography. One of the most common methods of image steganography is LSB which can also be applied to video steganography. In this method, the least significant bits of the frames of host video are used to carry the secret information (Jagadeesh & Sivan, 2020; Chae & Manjunath, 1999; Provos & Honeyman, 2003; Choudry & Wanjari, 2015). As described in Edmead (2019), video steganography hides secret data into cover video. The application areas of video steganography change from the defense industry to copyright control. The main ground behind being the widespread usage of video steganography is due to fact that the people can’t distinguish little changes in digital video (Mstafa, Elleithy & Abdelfattah, 2017). It is now becoming more and more important because of two essential reasons. These are advances in computer science and the increasing importance of the security problem of any given network. The final word about the importance of the video steganography is related to the video itself. The video offers big sizes for data embedding and more sharing among people on the net when it is compared with other multimedia files such as music, image, and text. It can be said that video applications are environments that are very suitable for the secret data embedding algorithms due to their widespread use and large secret data embedding capacities (Suo et al., 2012; Khosla & Kaur, 2014).

Imperceptibility, robustness, and load are the three important parameters of image and therefore video steganography. Imperceptibility is directly related to the visual quality of the stego video and means a lower degradation rate. This means that a higher imperceptibility will not get the attention of the intruder (Liu et al., 2013; Liu et al., 2019). Robustness is the other parameter and related to the resistance of steganography against any attack such as statistical, signal processing, cropping, scaling *etc.* Video steganography with a higher level of robustness means to have secret message unchanged in the case where stego video is under attack (Liu et al., 2019). Embedding capacity, which is also called, payload means the ratio of the number of the embedded secret message bits to the total number of pixels of the cover image. What is desirable for video steganography is to embed as much as possible secret message bits without sacrificing imperceptibility. Thus, there must be a balance between imperceptibility and payload.

Liu et al. (2019) recommended classifying the video steganography algorithms into three techniques according to the embedding position of the secret data. In the pre-embedding technique, the secret data is embedded in raw domain video (uncompressed one) by using methods of spatial and transformation domains. This technique allows the sender to implement embedding secret data to only uncompressed domain video. Unlike the pre-embedding class, the intra-embedding class makes use of compressed domain video to embed the data. It should be noted here that, the choice of levels of the video compressions such as intra-prediction, motion vector, *etc.* play an important role in the success of this technique. The last video steganography technique is the post-embedding technique that is based on embedding secret data in the bitstream of compressed domain video.

## Related Works

In Ramalingam & Isa (2014), researchers tried to improve the security of LSB in video steganography by introducing a sequential encoding and decoding-based video steganography. The method requires video preprocessing operation. The best PSNR ratio and MSE values were obtained as 71.52 dB and 0.01, respectively. At this best ratio, the payload was 0.11 bits per pixel (bpp). The advantage of the method is that it is simple to implement whereas the drawback of the method is less security. The studies (Kapoor & Mirza, 2015; Shinde & Rehman, 2015) divided secret messages into chunks of 2 bytes and embedded the 2 bytes of the message into the first two pixels of the frame starting from the first frame (1 byte each pixel). The best PSNR ratio and MSE values were obtained as 52.94 dB and 0.33, respectively. The payload was calculated as 2.66 bpp for all sizes of videos. Since only the first two pixels of each video frame were used for data hiding the method is quite vulnerable against statistical attacks. In another study, researchers proposed a steganography algorithm based on colour histograms for the data embedding into the video clips directly. The histogram constant value (HCV) is calculated by using all frames of the cover video. HCV is not only used for the selection of the frames in which the data will be embedded but also data embedding process. The selection of the frames is implemented by choosing the frames whose histogram variation is higher than HCV. The next stage is based on segmenting these frames into blocks to see the changes between successive pixels. The final step is to hide secret data into 3 LSB of each determined pixel. According to the hiding capacity (average of payload ratio is 1.1%), this method is restricted because it is only based on the HCV value. The average PSNR ratio was obtained as 48.84 dB (Kelash et al., 2013; Mstafa & Elleithy, 2016a). In the study implemented by Chitra & Thoti, (2013), they proposed a technique that takes eight bits of the data at a time and conceals them in LSBs of RGB (Red, Green, and Blue) pixel values of the carrier frames in 3, 3, 2 order respectively. The authors obtained the best PSNR and MSE values as 53.04 dB and 0.32, respectively. The authors claimed that payload was obtained as 2.66 bpp for different sizes of videos. Since the method doesn’t include any algorithm to choose a frame and the pixels for data embedding the method is vulnerable against attack when the imperceptibility is no longer valid. In Dasgupta, Mandal & Dutta (2012), a hash based LSB technique has been proposed. Eight bits of the secret information are divided into 3, 3, 2 and embedded into the RGB pixel values of the cover frames respectively. The authors obtained the best PSNR and MSE values as 45.67 dB and 0.34, respectively. The payload was obtained as 2.66 bpp for all sizes of videos. The method used in this study is almost the same as the method used in Chitra & Thoti (2013). Thus, the criticism made for the security in Chitra & Thoti (2013) is valid for this study. Gupta & Chaturvedi (2013) proposed an advanced approach for dynamic data protection using LSB and a hybrid approach. The proposed method for replacing one or two or three LSB of each pixel in the video frame and applying Advanced Encryption Standard (AES). The problems of security and high volume of secret data embedding are tried to be overcome by the authors. For 1-bit LSB substitution & AES, they obtained PSNR as 49 dB whereas for 3-bit LSB&AES they obtained PSNR as 35 dB. Even the security of the system is tried to be increased with AES, the payload is only 1 bpp for 1-bit LSB substitution that has the highest PSNR value. Additionally, the secret data is embedded from the first pixel of the frame in a sequential manner. All these problems make the method maintain the trade-off among imperceptibility, robustness, security, and payload. In Yadav, Mishra & Sharma (2013), researchers used a new video steganography algorithm that embeds a secret video into cover video. The first step of the method is converting frames of secret video into 8-bit binary values. Later, encryption with XOR is implemented on these converted frames. The final step is to embed encrypted frames into the LSB of each frame of the cover video by sequential encoding. The payload is 1 bpp. The highest PSNR value was obtained as 37.76 dB. The method has low security due to simple secret key and sequential encoding. Younus & Younus (2019) showed the secret message was encrypted, and then the knight tour algorithm was utilized for selecting the pixels inside the randomly selected frame was utilized for embedding the secret message inside it with the LSB method. The authors claimed that in terms of quality the proposed method has a superior performance compared to the previous steganography methods, with a high PSNR of 67.3638 dB and the lowest MSE of 0.2578. The payload of the method was 2.92 bpp. Selecting frames randomly and making use of a simple encryption method makes the method be vulnerable against statistical attacks. Dasgupta, Mandal & Dutta (2013) proposed a hybrid video steganography scheme for efficient, and effective information embedding. The authors used the method they developed in Dasgupta, Mandal & Dutta (2012), that is a 3-3-2 LSB-based scheme, and enhanced it using a Genetic Algorithm (GA) which aims to get an optimal imperceptibility of hidden data. Experimental results showed a substantial improvement in the PSNR (41.613 dB) and MSE (0.99) values (after optimization) over the base technique. PSNR value they obtained was worse than the PSNR value obtained from Dasgupta, Mandal & Dutta (2012). They have still the same payload (2.66 bpp) as in Dasgupta, Mandal & Dutta (2012). Both studies have lower PSNR and once the secret data is recognized it is easy to extract due to 3-3-2-LSB based embedding scheme.

Khupse & Patil (2014) proposed a method that is based on the region of interest (ROI) to hide the secret message. The idea behind the algorithm is to seek and find the skin region of the cover video frame to embed secret data in it. The preprocessing has been implemented on cover video frames. After finding ROIs in cover frames the frames are converted to YCbCr colour space before embedding of secret data takes place. The frame having the least MSE is selected to embed secret data. The authors obtained a PSNR value as 85.18 dB. Since only one frame is used for data embedding the payload is very low (2,120 bits per video). Because the method only makes use of one frame with the lowest MSE values, it has a very limited payload and low security. Apart from the selection of the frame with the lowest MSE, the method seems to be image steganography since only one frame is used for embedding. In other studies (Mstafa & Elleithy, 2016b), the authors combined the Kanade-Lucas-Tomasi (KLT) tracking and Hamming codes. The method includes encryption of the secret data with Hamming codes, determining the facial regions as ROI by KLT and then implementation of data embedding with adaptive LSB method. The authors claimed that they obtained PSNR and payload as 74.54 dB and 1 bpp for 1 LSB method. But, the Bit Error Code (BER) and Similarity SF values are reasonable but not ideal when statistical attacks such as Gaussian white, Median filtering, Salt & Pepper, *etc.* were applied. The other drawback of the method is being easily breakable once somebody understands that the faces in the images are used for embedding secret data.

All the studies mentioned till this point are not resistant against signal processing, noises, and compression. But following studies have claimed to be resistant against some video compression algorithms:

Sadek, Khalifa & Mostafa (2015) claimed that they proposed ROI based video steganography method that is resistant against compression. By using face detection and tracking algorithms the skins in the video frames are determined as ROIs. Then, the secret data is embedded into the blue and red colour channels of pixels of ROIs. The authors claimed that their proposed method is robust against one compression domain video (MPEG). But, it can be told that the both imperceptibility and payload they obtained are lower when compared with skin region based studies in literature (Khupse & Patil, 2014; Mstafa & Elleithy, 2016b). The obtained PSNR and payload are 54.64 dB and 0.23%, respectively. In Alavianmehr et al. (2012), the authors proposed histogram distribution constrained (HDC) based method video steganography. The method is applied on uncompressed domain video for embedding secret data. The stego video is called a watermarked video. The authors claimed that the method they proposed allows lossless secret data extraction and cover video unless the watermarked video is attacked by signal processing, noise, *etc.* According to the authors, their proposed method is resistant against H.246/AVC video compression. But this let the method sacrifice imperceptibility and payload since PSNR and payload values were obtained as 36.97 dB and 1.34%, respectively. Noda et al. (2004) proposed a method based on wavelet compression for video data and bit-plane complexity segmentation (BPCS) steganography. 3-D set partitioning in hierarchical trees (SPIHT)-BPCS steganography and MotionJPEG2000-BPCS steganography are presented and tested, which are the integration of 3-D SPIHT video coding and BPCS steganography, and that of Motion-JPEG2000 and BPCS, respectively. The one LSB-plane and the two LSB-planes were used to embed data for the number of bit-planes 11 and 12, respectively. They found that 3-D SPIHT-BPCS is superior to Motion-JPEG2000-BPCS with regard to embedding performance. The authors claimed that their method is robust against 3D–SPIHT and Motion-JPEG2000 compression. This robustness value depends on average payload and almost acceptable imperceptibility. The average of payload ratios are 18% for 1 bit-plane and 28% for 2 bit-planes. Average PSNR values are 43.55 dB for 1 bit-plane and 42.55 dB for of 2 bit-planes. When these values are considered it can be easily told that even for a one-bit plane their method has low imperceptibility and an almost acceptable payload. Due to low PSNR values for both planes stego video can get the attention of the intruder. Once this happens it would not be difficult to extract the secret data since the method is based on Discrete Wavelength Transform (DWT) and Bit-Plane Complexity Segmentation (BPCS) steganography.

As can be seen from the studies mentioned up to now and listed in Table 1, the struggle is to have a video steganography method that has a trade-off among robustness, imperceptibility, and payload. Robustness is related to the resistance of a cover video against signal processing, noises, physical deformation, compression, and statistical attacks. Imperceptibility is related to having an almost identical cover and original videos. Payload is related to embedding as much as secret information into cover video without scarifying imperceptibility and robustness. When these points are considered, it will be seen that none of the studies listed in Table 1 provides these trade-offs among four parameters. That’s the reason for ongoing studies in the raw domain of video steganography; to have a good balance among these three parameters. This point also motivated us to contribute to the gap of the balance of robustness, payload, and imperceptibility parameters of video steganography. As can be seen from Table 1, the proposed method has the highest PSNR ratio (80.01dB) and one of the highest payload (7.5%). Since Khupse & Patil (2014) only used one video frame for data embedding like image steganography making a comparison with the PSNR value of their study isn’t significant. The average PSNR and payload values of the proposed method were calculated as 71.13878 dB and 14.25%. These values are still among the highest ones and prove the imperceptibility of the proposed method. Additionally, the proposed method offers security in such a way that it is impossible for a third party to extract the secret message from the cover video unless the intruder has 18 previously created sub-pattern blocks that form KBM. Moreover, extra security is provided by letting shifting options in four directions (up-down-right-left) and rotation options up to 270°for KBM. XOR and AND logical operations are not only used for choosing directions/degree of this rotation but also for choosing which of the 64 × 64 video blocks will be chosen to embed the secret data as the next block. The ability to choose different channels of the RGB of a pixel to embed the secret data adds an extra level of security. Last but not least, the non-sequential data embedding in all frames algorithm has the possibility to embed one character within the first frame and the next character within the last frame. All these aspects ensure the high-level of security of the proposed video steganography method.

**Table 1:** Comparison of the proposed method with surveyed video steganography techniques.

**Studies**	**PSNR (dB)**	**Payload (bpp/%)**	**MSE**	**Robustness**
**The proposed method**	**Best value 80.01 average 71.13878**	**For best PSNR value 7.5% average 14.25%**	**Best value 0.00066 average 0.9996**	**Not robust enough against compression and noise but robust enough against physical operation and visual attack, provides higher security**
Ramalingam & Isa (2014)	Best value 71.52	Best value 0.11	Best value 0.01	Not robust enough against signal processing, noise, physical operation, statistical attacaks and compression
Kapoor & Mirza (2015)	Best value 52.94	Average value 2.66	Best value 0.33	Not robust enough against signal processing, noise, physical operation, statistical attacaks and compression
Kelash et al. (2013)	Average value 48.84	Average value 1.1%	—	Not robust enough against signal processing, noise, physical operation, statistical attacaks and compression
Chitra & Thoti (2013)	Best value 53.04	Average value 2.66	Best value 0.32	Not robust enough against signal processing, noise, physical operation, statistical attacaks and compression
Dasgupta, Mandal & Dutta (2012)	Best value 45.67	Average value 2.66	Best value 0.34	Not robust enough against signal processing, noise, physical operation, statistical attacaks and compression
Gupta & Chaturvedi (2013)	Highest value 49	Average value 1	—	Not robust enough against signal processing, noise, physical operation and compression but robust against statistical attacks
Yadav, Mishra & Sharma (2013)	Highest value 37.76	Average value 1	—	Not robust enough against signal processing, noise, physical operation, statistical attacaks and compression
Younus & Younus (2019)	Highest value 67.3638	Average value 2.92	Lowest value 0.2578	Not robust enough against signal processing, noise, physical operation, statistical attacaks and compression
Dasgupta, Mandal & Dutta (2013)	Average value 41.613	Average value 2.66	Average value 0.99	Not robust enough against signal processing, noise, physical operation, statistical attacaks and compression
Khupse & Patil (2014)	Average value 85.18	Low payload only frame is used (2120 bits per video)	—	Not robust enough against signal processing, noise, physical operation, statistical attacaks and compression
Mstafa & Elleithy (2016b)	Average value 74.54	Average value 1	—	Not robust enough against signal processing, noise, physical operation, statistical attacaks and compression
Sadek, Khalifa & Mostafa (2015)	Average value 54.64	Average value 0.23%	—	Robust against MPEG-4 codec
Alavianmehr et al. (2012)	Average value 36.97	Average value 1.34%	—	Robust against H.264/AVC codec
Noda et al. (2004)	Average value 42.55	Average value 18%	—	Robust against 3D–SPIHT and Motion-JPEG2000 compression

**Note.**

The proposed method is shown in bold.

When it comes to resistance against noise and transformation-based attacks to cover video the proposed method also has contributions to fill the gap. To ensure that the hidden data is affected by the negative factors that may cause a data container on the cover video, with the least loss, an equal amount of data is hidden in each video frame with the sequential data embedding in all frames and the non-sequential data embedding in all frames algorithm that are two of 12 different embedding schemes of the proposed method. When these aspects are considered, it can be said that the proposed method has robustness superior to other methods. The drawback of the proposed method is to have no robustness against compression and noise. This is still acceptable since it offers high imperceptibility and payload with partial robustness.

## Material and Methods

### KBM

**Figure 1:**
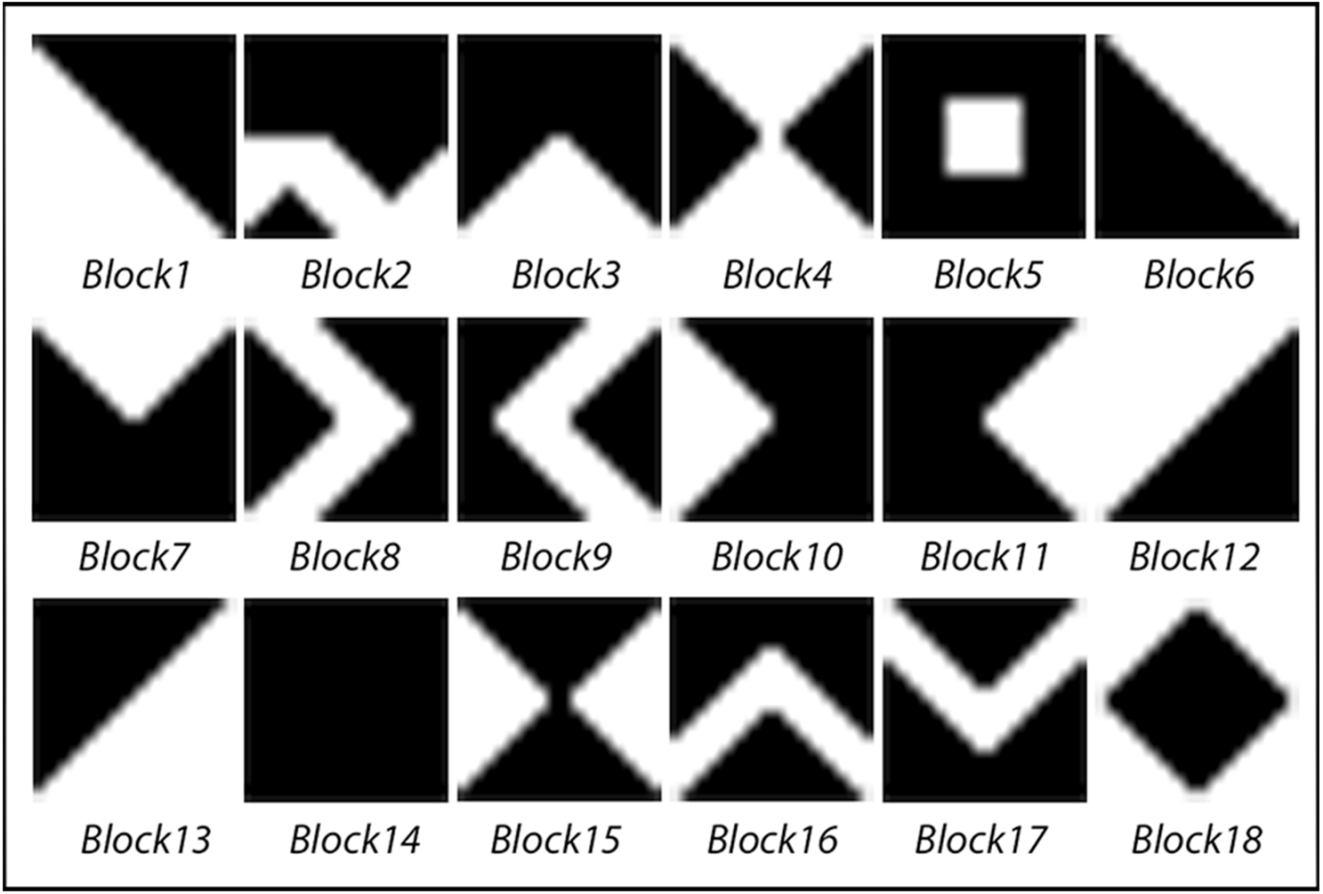
Eighteen pattern blocks.

**Figure 2:**
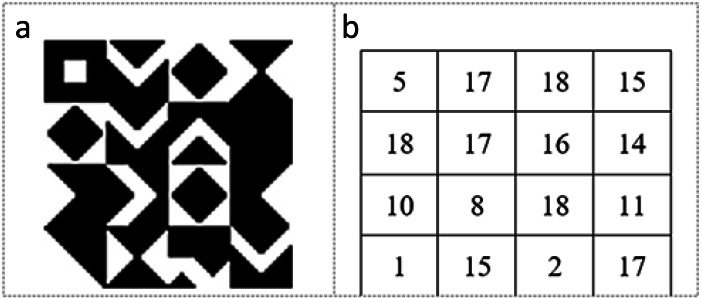
(A) Sample KBM. (B) The order of the selected sub-pattern blocks.

The KBM is created to determine the data embedding areas in the video frame. It consists of a 64 × 64 pixels main pattern block that includes 16 sub-pattern blocks in 16 × 16 pixels. The 16 sub-pattern blocks are randomly selected from 18 previously created sub-pattern blocks that are given unique numbers and can be seen in Fig. 1. Figure 2A shows a sample of KBM whereas Fig. 2B shows the ordering of the 16 sub-pattern blocks selected to form sample KBM in Fig. 2A.

**Figure 3:**
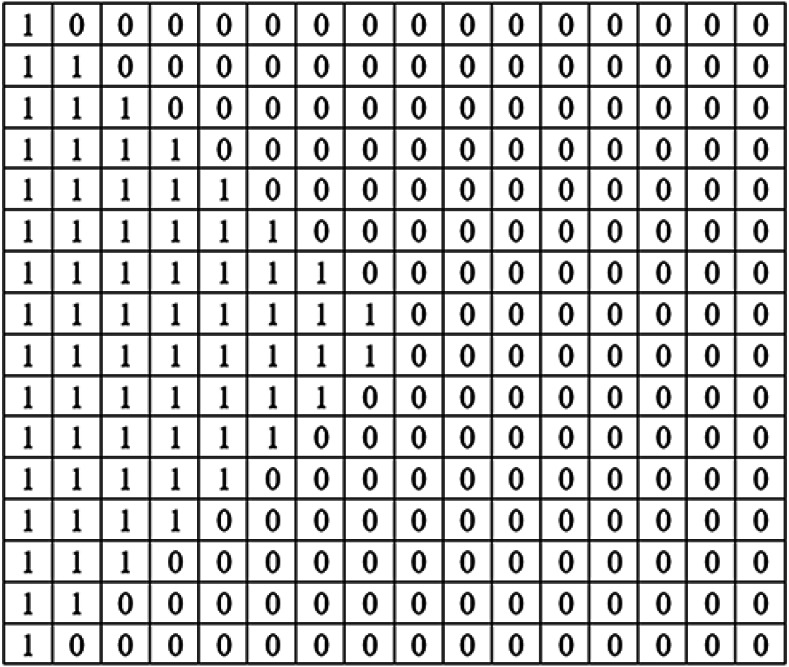
The 16 ×16 sub-pattern block matrix structure of sub-pattern block number 10.

Coding of KBM is implemented by using 0 for areas corresponding to the black colour (0) value and 1 for areas corresponding to the white colour (255) value in KBM. The areas represented by 0 within the KBM are used as closed areas where the data cannot be embedded while the areas represented by 1 are used as open areas suitable/appropriate for the data embedding. Figure 3 shows how one of the sub-pattern blocks of sample KBM in Fig. 2A is coded.

#### Fixed block structure

In algorithms using the fixed block structure, the same KBM is used for each 64 × 64 pixel block area of the video frame. The KBM includes pixel areas that are suitable for the data embedding as mentioned before.

After the completion of the data embedding process by using a KBM in the first 64 × 64 pixel block area of the video frame, the same KBM is used in the next 64 × 64 pixel block areas of the video frame. The processes continue until the whole data is embedded within the video frames.

#### Variable block structure

In algorithms using a variable block structure, the first placement of the KBM into the video frame is the same as in the fixed block structure. After the completion of the data embedding process performed by using a KBM in the first 64 × 64 pixel block area of the video frame, the KBM will be changed for the next 64 × 64 pixel block area of the video frame. This change is implemented according to the colour values of the most recent data-hidden pixel-which is the last appropriate pixel for the data embedding within the 64 × 64 pixel block area of the current video frame. Shifting, rotating, or shifting-rotating operations in the KBM depending on the most recent data-hidden pixel are carried out as follows:

• If all the RGB colour channels of the most recent secret data-hidden pixel are used to embed the data then B and G colour channel values of the related pixel and
• If only one channel of RGB colour channels of the most recent data-hidden pixel is used to embed the data then the values of colour channels in which no secret data is embedded will be used for changing the structure of the KBM.

Let us make clear the variable block structure by giving an example. The process steps for the determination of the next KBM structure (movement processes) are as follows for sample values:

***Step 1.*** The colour values of the last and most recent secret data-hidden pixel within the current block are read. Assume that these values are *B* = 178, *G* = 205 and *R* = 114

***Step 2.*** The first LSB is the bit where the data is embedded. The second LSB represents the control bit and the third, fourth, fifth, and sixth LSB values represent the number of steps for related operation. Assume that each RGB colour channel is used for the data embedding. The colour channels B (ch1) and G (ch2) are used as control and step indicators for left–right (B) and up–down (G) block movements, respectively.

 B = ch1 = 10110010   G = ch2 = 11001101
 For ch1 of pixel value,
 178 = 10 1100 1 0
 Embedded secret data (LSB) = 0
 For control bit (ch1_control) = 1
 For step number (ch1_step) = (1100)_2_ = 12-pixels is the calculated value.
 For ch2 of pixel value,
 205 = 11 0011 0 1
 Embedded secret data (LSB) = 1
 For control bit (ch2_control) = 0
 For step number (ch2_step) = (0011)_2_ = 3-pixels is the calculated value.

***Step 3.*** In the directions determined by the control parameter, the 16 sub-pattern blocks that make up the KBM are moved inside KBM as much as the calculated number of pixels. The reader is kindly requested to accept the KBM movement expression as the movement of the 16 sub-pattern blocks in the KBM from now on. As a result of these movements, the structure of the next KBM is determined.

***For the shifting process:***

 For ch1_control = 0 or 1,
 0 means shifting right process,
    1 means shifting left process.
 ch1_step = number of steps in pixel regarding to shift right or left
 For ch2_control = 0 or 1,
    0 means shifting up process,
    1 means shifting down process.
 ch2_step = number of steps in pixel regarding to shifting up or down

***For the rotating process:***

 For (ch1_control AND ch2_control) == 0 or 1 value,
    0 means rotating right process (90^∘^)
    1 means rotating left process (90^∘^)
 ch1_step = number of steps in pixel regarding to rotating right
 ch2_step = number of steps in pixel regarding to rotating left
 Since the KBM will be equal to itself after every 4-steps rotation, the shifting process is implemented with modulo 4 operation.
    ch1_step = ch1_step % 4 or
    ch_2step = ch2_step % 4

***For the shifting-rotating process:***

 Assuming that the movement selection for the determination of the KBM is ”shifting-rotating”, according to the sample values.
 For the values of ch1_control = 1 and ch1_step = 12, the KBM will be shifted 12 steps in the pixel in the left direction.
 For the values of ch2_control = 0 and ch2_step = 3, the KBM will be shifted 3 steps in the pixel in the up direction.
 For (ch1_control and ch2_control) == 0
   ch1_step = 12
   ch1_step = ch1_step % 4 values, the KBM will be rotating 0 step in the right direction.

The process to be followed where only one channel of RGB is used for the data embedding includes similar steps for changing the KBM. In this case, the colour channels in which no secret data is embedded will be used as control and step indicators for left–right and up–down block movements.

### The data embedding into video frame

As mentioned previously, the process of embedding the data in the determined 64 × 64 pixel block areas of the video frame is performed based on the KBM values. To increase the security of the system, one more logical operation is added for the data embedding process. The data is not embedded directly to the appropriate pixel determined by the KBM. The colour values of the most recent secret data-hidden pixel within the 64 × 64 pixel block area in the video frame determine whether the data will be embedded in the next pixel or no. It should be indicated here that the movement, control, and embedding operations are implemented by using each of the 16 × 16 sub-pattern block and related block area of the video frame. Let us assume that data embedding is in the progress and one of the 16 sub-pattern blocks of KBM is being used for this aim. If it is required to go deeper and explain movement and controls in detail it will be as follows.

Assume all the RGB colour channels are being used for the data embedding by the user. Then, the colour channels to be used for the control values are kept in the variables ch1 (B) and ch2 (G). Thus, the direction of movement within the related sub-pattern is determined as horizontal or vertical after the colour channels ch1 and ch2 have been subjected to the XOR operation and the result obtained has been subjected to the AND logical operation:

    If (XOR (ch1) and XOR (ch2)) == 1
    Direction == Horizontal
    If (XOR (ch1) and XOR (ch2)) == 0
    Direction == Vertical
 Whether the data can be hidden to the pixel reached in the direction of move is determined after the colour channels ch1 and ch2 are subjected to the XOR operation and the result obtained is subjected to the OR logical operation.
    If (XOR (ch1) or XOR (ch2)) = = 0
    Situation == False
    Situation = False indicates that the data cannot be hidden within the pixel reached.
    If (XOR (ch1) or XOR (ch2)) = = 1
    Situation == True
    Situation = True indicates that the data can be hidden within the pixel reached.

If the pixel is found as appropriate for the data embedding after logical operations and the corresponding member value of this pixel in the related sub-pattern block of KBM is 1, then the data embedding process is performed within this pixel. If the pixel is not found as appropriate for the data embedding after logical operations, it is moved on to the next member in the related sub-pattern block of KBM towards the direction of movement. The value of this member is checked to see the data embedding appropriateness (0-1). The movement, control, and embedding operations are implemented until all the rows of the related 16 × 16 sub-pattern block are scanned. Within the 16 × 16 sub-block, the movement takes place in two directions, from left to right or from top to bottom. It means that when the horizontal movement is in progress the end of any row means to jump the first member of the next row including the final row for control. Similarly, when vertical movement is in progress members in the next row of the related column will be visited. Termination of the movement or search in the 16 × 16 sub-pattern block of KBM will be implemented as follows:

(i) When the movement direction is vertical, and search is in the final row and
(ii) When the movement direction is horizontal, and the final member of the last row is reached.

After the completion of the movement, control, and data embedding processes for the first 16 × 16 pixel block in the video frame, the 2nd 16 × 16 pixel block (on the right) is checked for the data embedding suitability by using the 2nd 16 × 16 sub-pattern block. The first member (0, 0) of the 2nd 16 × 16 key sub-pattern block is the starting point of the new search and movement direction inherited from the previous key sub-pattern block. This operation is repeated for 16 sub-pattern blocks that correspond to 16 numbers of 16 × 16 pixel blocks of the related frame. Assume that the 64 × 164 KBM in Fig. 1 is used for the data embedding. In this case, the order of searching or visiting the 16 sub-pattern blocks will be 5-17-18-15-18-17-16-14-10-8-18-11-1-15-2-17. After the completion of the control and the data embedding processes in the 64 × 64 pixel block area of the video frame, control and movement processes are re-started for embedding the data in the second 64 × 64 pixel block area of the video frame. This is implemented by using KBM. The new KBM can be the same, shifted, rotated, or shifted-rotated in the structure for the next 64 × 64 pixel block area of the video frame depending on the user preference. The default values of the parameters for moving in the new KBM is as follows:

(i) Start from the first 16 × 16 sub-pattern block at the top left corner of KBM.
(ii) The direction to go for the next 16 × 16 sub-pattern block is from left to right (Direction == Horizontal).
(iii) The data embedding for the first pixel in the next sub-pattern block is allowed (Situation==True).

At this point, it is important to mention the choice of the video frames for the data embedding. The proposed method offers three options to the user. These are sequential data embedding, sequential data embedding in all frames, and non-sequential data embedding in all frames algorithms. In the sequential data embedding algorithm, the data in the binary form is embedded into the video frames starting from the first frame and progressing respectively, based on the selections of colour the channel (RGB, R, G, or B) to embed the data and the fixed or variable (shifting, rotating or shifting-rotating) block structure. Subsequent frames are used until all the data is embedded. Thus, all the frames of the video will not be used for the embedding process.

In the algorithm of sequential secret data embedding in all frames, ”pixels suitable for data embedding” are determined within the video frames by checking all frames sequentially starting from the first video frame in line with the direction of movement and controls as mentioned in the previous paragraphs. The appropriate pixels are kept in a sequence. The number of bits to be hidden within each video frame is determined. After the determination of the pixel areas where data will be hidden, the data in binary form are placed sequentially in the pixel areas that are indicated by the index values suitable for embedding secret data, starting from the first video frame using the LSB method. Thus, all the video frames are used for the data embedding.

In the non-sequential data embedding, the data is non-sequentially embedded in all frames according to the selections of the KBM and the colour channels for data embedding. The determination of the pixels to embed the data is calculated as in the process of the sequential data embedding in all frames. In the data embedding process, the data is embedded within the frames non-sequentially by using randomization. In this case, it is possible that one character of the data is embedded within the first frame and the next character is embedded in the last frame. This will increase the security of the proposed method.

Since the sequential data embedding in all frames and the non-sequential data embedding in all frames algorithm will allow an equal amount of data embedding in each video frame the data loss will be less in transformation operations. The lost data can be easily obtained from the entire text with natural language processing.

### Embedding of control values

In the data embedding process, information regarding the selected colour channel, the selected message’s length, the selected structure (including order of selected 16 sub-pattern blocks) and type of KBM (fixed, shifting, rotating or shifting-rotating), and the selected algorithm (the sequential data embedding, the sequential data embedding in all frames or the non-sequential data embedding in all frames) are embedded within the stego object as control values to extract the secret data at the receiver side. The last 64 × 64 pixel block area of each video frame is used to hide these key control values. There is extra security here as explained in the following lines.

Following logical operations are applied for the colour channels’ bit values of every pixel of 64 ×64 block area:

 XOR (B_7−1_) AND XOR (G_7−1_) AND XOR (R_7−1_) == 0 indicates an inappropriate pixel for embedding the data.
 XOR (B_7−1_) AND XOR (G_7−1_) AND XOR (R_7−1_) == 1 indicates an appropriate pixel for embedding the data.

where (B_7−1_) represents 7 MSBs of the B channel, (G_7−1_) represents 7 MSBs of the G channel and (R_7−1_) represents 7 MSBs of the R channel.

If the condition is met by pixel control value is embedded to related pixel. Control values are hidden within the sequential frames, starting from the first video frame. After each control value is hidden sequentially, a video frame is left empty, and the embedding process is continued in the following video frame.

### System structure

**Figure 4:**
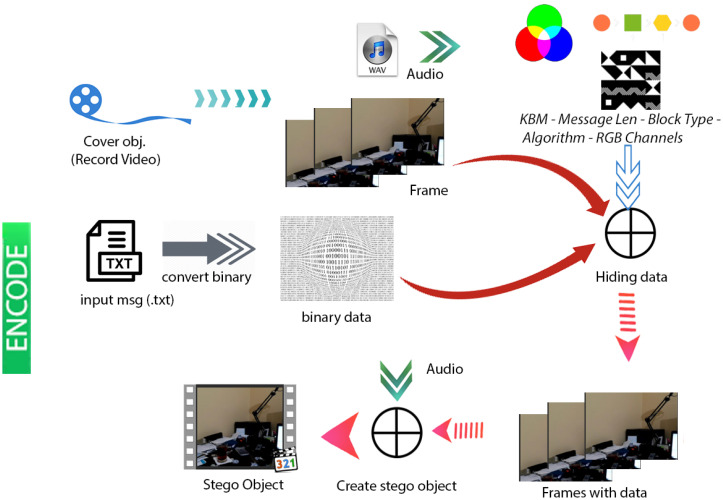
Video steganography encode structure.

**Figure 5:**
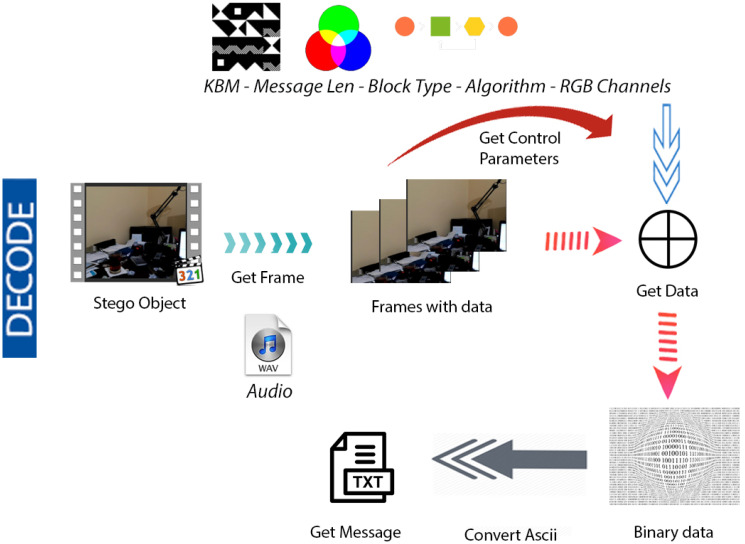
Video steganography decode structure.

Figures 4 & 5 show the proposed video steganography encode and decode structure. The system mainly consists of the processes of creating the stego object (encoding) and obtaining the hidden message (decoding). As can be seen from Fig. 4, dynamically shot video (cover object) is separated into frames and audio files in the decoding process. The secret message is embedded into the frames by using KBM. Control values namely structure and type of KBM, message’s length, algorithm, and colour channel are embedded into the last 64 × 64 pixel block area of each frame. The final step is a combination of frames and audio files to form the stego object. When it comes to decode structure shown in Fig. 5 the operations are almost reversed. The first stego object is separated into frames and audio files. The control values are read from the last 64 × 64 pixel block areas of frames. By using KBM and other control values the secret message is obtained. The steps of the encoding and decoding process are explained in detail below.

#### Creation of the stego object – encoding

**Figure 6:**
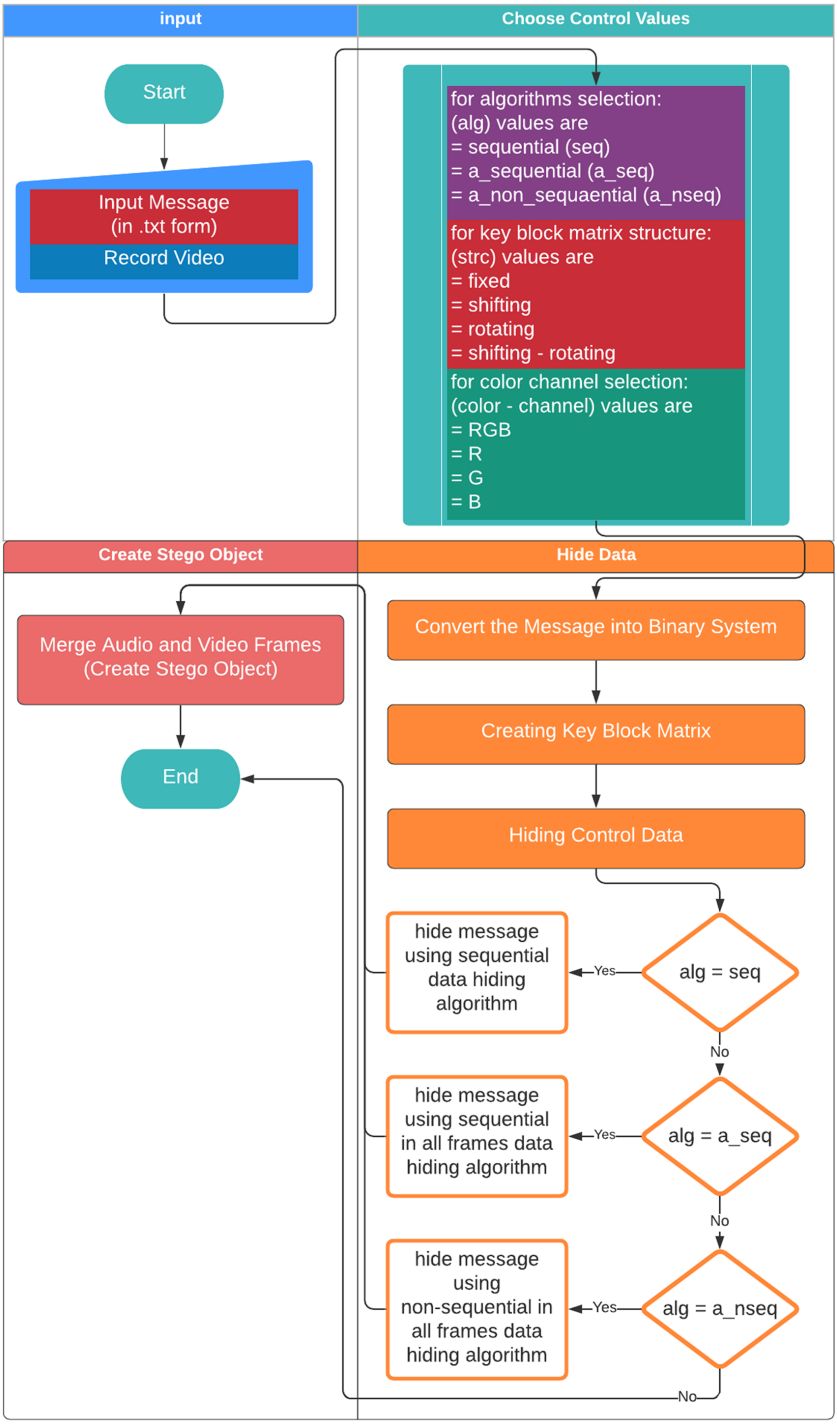
Flowchart on the creation of the stego object.

Figure 6 shows the flowchart displaying the creation of the stego object based on the proposed method. First, the user gives a name to the .txt file including the secret message to be hidden. Video recording is made for the cover object to be used in the creation of the stego object. The dynamically produced cover object is created through the user recording a momentary image with a camera. The video frames and audio files are recorded separately to be combined afterwards. Each video frame is given a number based on the order of recording in the folder and saved as .png while the audio file is saved as .wav. The user chooses the methods used to generate the stego object. First, the user selects the algorithm to be used in the data embedding process among algorithms namely the sequential data embedding, the sequential data embedding in all frames, and the non-sequential data embedding in all frames. Then, 16 sub-pattern blocks and type of KBM (fixed, shifting rotating, or shifting-rotating) are selected. Afterwards, the colour channel to embed the data is selected among RGB, R, G, or B. The ASCII data input by the user in the .txt (text) format is converted into the binary system. Then, the embedding process is implemented according to those selected parameters.

The steps of the process of embedding sample data within a dynamically recorded video are as follows:

***Step 1.*** The secret message is entered.

Let’s assume that the message has a size of 42.8 kb.

***Step 2.*** Dynamic video recording is performed as the cover object.

The dynamically recorded video has a pixel size of 640x480 and consists of 330 video frames.

***Step 3.*** The selection of the control values is performed.

Assume that the selections are as follows:

 The data embedding algorithm selection: “The Sequential Data Embedding” algorithm,
 Block structure selection: The shifting-rotating block structure,
 colour channel selection: The “RGB” colour channel.

***Step 4.*** After the completion of the data input processes, the input message is converted into a binary code and the message length is calculated. The message, that is recorded as ASCII, is kept in a .txt file by being converted into a binary code. The sample message length is calculated as 376193 bits. This value is included in the control values.

***Step 5.*** A KBM is created depending on the ordering of selected 16 sub-pattern blocks. This KBM is included in the control values.

***Step 6.*** To obtain the secret message at the receiver side, the information regarding the KBM, colour channel, message length, and the data embedding algorithm are embedded within the video frames as control values.

***Step 7.*** Based on these selections, the data embedding process is performed.

**Figure 7:**
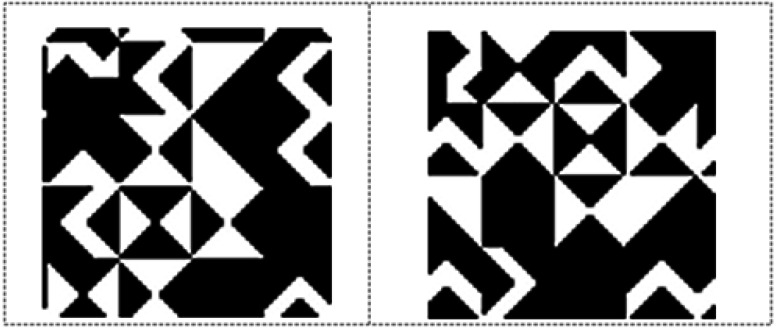
11th and 12th 64‘× 64 KBM structures for the 1st video frame.

**Figure 8:**
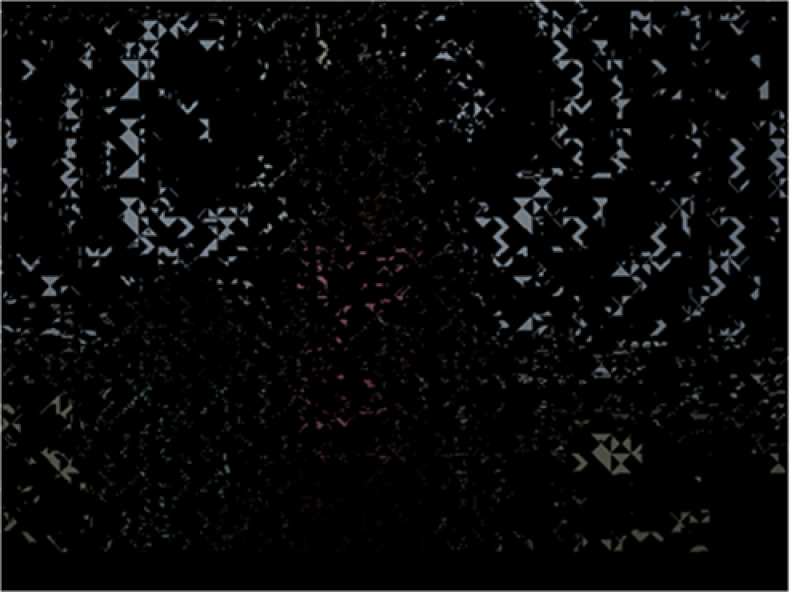
Data-hidden areas within the 2nd frame.

Figure 7 shows the 11th (left) and 12th (right) KBMs produced depending on the selection of KBMs structure and type (shifting-rotating block) for the 1st video frame. As can be seen from Fig. 7, 12th KBM is shifted and rotated version of 11th KBM. Figure 8 shows the areas where data was hidden in the 2nd video frame. The number of frames that constitute the video and their resolution values are used to calculate the maximum capacity of data to be hidden in the video.

The data to be hidden are placed in the fields obtained by dividing the pixels of the video frame into 64 × 64 bit blocks. The last block fields parsed for each frame are used to hide control data. The number of blocks in which data can be hidden within the video frame is determined as follows:

For a (*w*) x (*h*) video:

(1) \begin{eqnarray*}No of horizontal blocks (nhb)=int(w/64)\end{eqnarray*}

(2) \begin{eqnarray*}No of vertical blocks (nvb)=int(h/64)\end{eqnarray*}

(3) \begin{eqnarray*}Total number of blocks (tnb)=nhb x nvb.\end{eqnarray*}

where *’w’* and *’h’* represents width and height of the video frame in pixel, respectively. The last 64 × 64 bit block areas of the frames where data can be hidden are used as areas reserved for hiding the control values. In this case, the total number of blocks to hide data for each video frame is:

(4) \begin{eqnarray*}tnb\text{_}final=tnb-1\end{eqnarray*}

The number of blocks in which data can be hidden for each frame of a sample 640 × 480 p video is calculated as follows:

 *nhb* = *int*(640/64) = > *nhb* = 10
   *nvb* = *int*(480/64) = > *nvb* = 7
   *tnb* = 10*x*7 = 70*blocks*.
   *tnb*_*final* = 70 −  − 1*blockperframe*.

Maximum number of bytes that can be embedded into the cover object regarding to the usage of last bit of each channel of an RGB pixel;

(5) \begin{eqnarray*}Capacity (RGB)=(number of frames x tnb x 64 x 64 x 3)/8(bytes)\end{eqnarray*}

Maximum number of bytes that can be embedded into the cover object regarding to the usage of only one colour channel of the pixel of an RGB image;

(6) \begin{eqnarray*}Capacity (R, G or B)=(number of frames x tnb x 64 x 64 )/8(bytes)\end{eqnarray*}

The capacity of the video file must be at least twice that of the secret message since KBM has black regions that correspond to pixel in which no data can be embedded. The proposed approach aims to embed secret text messages into the video. When the user would like to embed video or audio messages by the proposed approach, the related file format must be converted into binary format. Later on, depending on the length of the converted file (bytes), the cover video must be shot. The length of the cover video t in time (second) can be calculated for a (*wxh*) video by using Eqs. (6) and (7).

(7) \begin{eqnarray*}t=converted file length (bytes)/Capacity (R, G or B)\end{eqnarray*}

The sizes of encoded and decoded secret messages are presented in Table 2. This table shows that the proposed method is a lossless video steganography method. Table 3 includes the times it takes for encoding and decoding each of secret message with respect to 3 different algorithms. Considering the above equations, it should be noted that as the size of the secret data presented in Table 2 grows, the encoding process that is calculated by Eq. (7) takes longer as can be seen in Table 3. Thus, depending on the time-cost efficiency, power of the computer and the requirements, the user is free to choose the secret message type (video, music or text) and size.

**Table 2:** Secret and extracted messages.

		**Encoded**	**Decoded**
1st secret message	Message length	376193 bits	376193 bits
	Message size	42.8 kb	42.8 kb
2nd secret message	Message length	1061049 bits	1061049 bits
	Message size	109 kb	109 kb

**Table 3:** Encoding and decoding times.

		**Sequential**	**All frame sequential**	**All frame non-sequential**
1st secret message	Decode	950 s	2745 s.	5920 s.
	Encode	1200 s.	3050 s.	6290 s.
2nd secret message	Decode	1628 s.	3720 s.	6420 s.
	Encode	1940 s.	3058 s.	3755 s.

#### Obtaining the secret message – decoding

For the decoding operation, the user is initially asked to enter the name of the stego object in the .avi file format as the input parameter. The input stego object is divided into frames. As the next step, the control values hidden within the last 64 × 64 pixel block areas of the video frames are obtained. The control values are read according to the order of embedding in the video frames. The information regarding the selected colour channel, the selected message’s length, the selected structure and type of KBM, and the selected algorithm is extracted. Finally, the secret data is extracted sequentially from the first video frame using control values and KBM. The LSBs of the related RGB colour channels of each pixel in which the data is embedded are read. All the data is saved in a ”.txt” file in binary format that will be converted into ASCII characters in the same file.

### Video quality metrics

Video quality is of importance in terms of the transmission, use and assessment of video. Video quality is a measurement of video distortion, which is perceived by subjecting a video to a certain processing system and comparing it with the original file (Akramullah, 2014). Video processing systems may cause certain disturbances in video signals that may affect the perception of the system or the user negatively. In the assessment of video quality, subjective quality assessment metrics evaluating the visual quality and content subjectively, or objective quality assessment metrics including the use of mathematical models based on the statistical values of video signals are used.

Objective video quality assessment models are mathematical models that allow for the approximation of subjective quality assessment results to be obtained from observers who are asked to rate the quality of a given video. In this context, the term model may refer to a simple statistical model or consist of complicated algorithms implemented in software or hardware (Akramullah, 2014).

Various metrics are used to approximate video quality. The most widely used metrics are PSNR, MSE, and SSIM. These metrics are based on a pixel-by-pixel comparison of source video signals and test video signals.

#### MSE

MSE is the mean of the squared difference between the corresponding pixel values of two images. It is an image quality evaluation based on the error sensitivity between the corresponding pixels. It gets an error value between 0 and 65025 for the 8-bit colour value (Shahid et al., 2015). This value is expected to be as small as possible for a high-quality video processing result. A value of 0 states that there is no difference between the two videos while 65025 represents the maximum difference for the 8-bit colour value. The closer the result is to 0, the more successful the video processing result is considered to be. The MSE value is calculated based on the following equation Eq. (8).

(8) \begin{eqnarray*}MSE= \frac{1}{n.m} \sum _{x=1}^{n}\sum _{y=1}^{m}({|}{f}_{(x,y)}-{f}_{(x,y)}^{{}^{{^{\prime}}}}{|})^{2}\end{eqnarray*}

where, *m* and *n* are the rows and columns of the cover image respectively, and *f*_(*x*,*y*)_ and ${f}_{(x,y)}^{{}^{{^{\prime}}}}$ means the pixel value at position *(x,y)* in the cover-image and the corresponding stego-image, respectively.

#### PSNR

PSNR is defined as the ratio between the maximum possible power of a signal and the noticeable power of the potential distortion (noise level in the video) in the processing and transmission of video. The logarithmic decibel scale (dB) is generally used for PSNR due to the wide dynamic range of signals. If the calculated PSNR value is close to 1, it indicates that the quality of similarity between two images is high. The PSNR value is calculated based on the following equation Eq. (9) (Haas et al., 2019).

(9) \begin{eqnarray*}PSNR=10.{\log \nolimits }_{10}( \frac{({f}_{x})^{2}}{MSE} )\end{eqnarray*}

where *f*_*x*_ represents the value of maximum pixel or maximum signal in the image. It is calculated as *f*_*x*_ = 2^*n*^ − 1 for the maximum signal value with *n* representing the pixel bit value. For example, pixel values are calculated as *f*_*x*_ = 255 for 8-bit colour values and as *f*_*x*_ = 1023 for 10-bit colour values.

#### SSIM

SSIM is a metric that displays the structural similarity between two images. In calculation, it uses structural distortion, perceptual structure and differences in luminance values. SSIM performs a comparison of the structural similarity between the original image and the processed image. The result of the comparison gives a value between 0 and 1. The quality and the similarity between the images increase as the result gets closer to 1. In the analysis process, SSIM takes local luminance and contrast into consideration. SSIM measures contrast, luminance and structural comparison using mean and variance values (Vranješ, Rimac-Drlje & Grgić, 2013).

For the SSIM value;

Luminance, contrast, and structural comparison functions are calculated based on the following equations, respectively.

(10) \begin{eqnarray*}I(x,y)= \frac{2{\mu }_{x}{\mu }_{y}+{C}_{1}}{{\mu }_{x}^{2}+{\mu }_{y}^{2}+{C}_{1}^{{}^{{^{\prime}}}}} \end{eqnarray*}

The luminance comparison function *l(x, y)* is a function of *μ*_*x*_ and *μ*_*y*_.*μ*_*x*_ and *μ*_*y*_ represents luminance of *x* and *y* signals and are estimated as the mean intensities. Constant *C*_1_ is included to avoid instability when ${\mu }_{x}^{2}+{\mu }_{y}^{2}$ is very close to zero.

(11) \begin{eqnarray*}c(x,y)= \frac{2{\sigma }_{x}{\sigma }_{y}+{C}_{2}}{{\sigma }_{x}^{2}+{\sigma }_{y}^{2}+{C}_{2}^{{}^{{^{\prime}}}}} \end{eqnarray*}

The contrast comparison *c(x, y)* is the comparison of *σ*_*x*_ and *σ*_*y*_. The standard deviation as an estimate of the signal contrast is used. But, in contrast comparison an unbiased estimate in discrete form (*σ*_*x*_, *σ*_*y*_) is used. *C*_2_ is a non-negative constant.

(12) \begin{eqnarray*}s(x,y)= \frac{{\sigma }_{xy}+{C}_{3}}{{\sigma }_{x}{\sigma }_{y}+{C}_{3}^{{}^{{^{\prime}}}}} \end{eqnarray*}

Structure comparison is conducted after luminance subtraction and contrast normalization. As in the luminance and contrast measures, a small constant *C*_3_ in both denominator and numerator is introduced.

When the three comparisons represented by Eqs. (10), (11) and (12) are combined to produce the similarity metric (SSIM) obtained from the signals x and y, the following Eq. (13) is obtained.

(13) \begin{eqnarray*}SSIM(x,y)=[I(x,y)]^{\alpha }[c(x,y)]^{\beta }[s(x,y)]^{\gamma }\end{eqnarray*}

where *α* > 0, *β* > 0 and *γ* > 0 are parameters used to adjust the relative importance of the three components.

## Applications

The Python PyCharm IDE was used in the coding process for the data embedding and analysis. Same video frames and audio files belonging to the dynamically recorded video were used for each application. The video has a pixel resolution of 640x480 pixels and 330 video frames. In the present applications, a 64 × 64 KBM consisting of the same pattern blocks was used for each application to ensure application reliability. To compare the data embedding processes, first, each colour channel was used separately, and the data embedding was performed under the LSB method. Afterwards, based on the selected algorithm structure; the data embedding processes are carried out by the LSB technique as the sequential data embedding, the sequential data embedding in all frames, or the non-sequential data embedding in all frames. The comparison of the original and stego frames was implemented based on the PSNR, MSE, and SSIM values. The results obtained are presented comparatively for each application in tables.

Table 2 shows the capacities of encoded and decoded secret messages for each application. As can be seen from Table 2 a lossless video steganography system is developed.

Table 3 shows the time takes to encoding and decoding secret messages for each application on a PC with an Intel Core i7 6500U 2.5 GHz microprocessor, 8 Gb RAM, NVidia GeForce 940 MX VGA and Windows 10 OS.

In the sequential data embedding algorithms, quality assessment is performed by considering only the number of frames including the secret message. Because this algorithm embeds the data sequentially from the first video frame throughout the subsequential video frames. Thus, the number of video frames in which the secret message is hidden varies based on the size of the secret message. For a reliable assessment of the results, only the frames used by this algorithm for the data embedding were used in the assessment process. Frames with no secret data were excluded from the assessment.

Since all frames are used by sequential data hiding and non-sequential data hiding in all frames the quality assessment is performed by considering all frames for these two algorithms.

## Results

Table 4 shows the quality metric results after two secret messages were sequentially embedded within the frames by the LSB method.

**Table 4:** LSB method quality metrics results for secret message 1 and secret message 2.

	**Message 1**			**Message 2**		
**Data hidden colour channel**	**MSE**	**SSIM**	**PSNR**	**MSE**	**SSIM**	**PSNR**
RGB	0.20397	0.99996	55.03516	0.28840	0.99739	54.49446
R	0.10196	0.99909	58.67726	0.14421	0.99840	56.57067
G	0.10166	0.99908	58.69619	0.14408	0.99839	56.57517
B	0.10185	0.99908	58.68525	0.14445	0.99840	56.56389

Tables 5 and  6 show the quality metric results obtained because of embedding each secret message within each colour channel based on the data embedding algorithms and KBM under the methods proposed.

**Table 5:** Quality metrics results for the first secret data.

**Block structure**	**Data hidden colour channel**	**The sequential data embedding**			**The sequential data embedding in all frames**			**The non-sequential data embedding in all frames**		
		**MSE**	**SSIM**	**PSNR**	**MSE**	**SSIM**	**PSNR**	**MSE**	**SSIM**	**PSNR**
Fixed	RGB	0.02576	0.99967	64.92870	0.00188	0.99998	75.40425	1,29654	0.99599	47.32575
	R	0.00938	0.99989	68.42653	0.00067	0.99999	79.97767	0.00067	0.99999	79.97940
	G	0.00898	0.99990	68.67953	0.00067	0.99999	79.95781	0.00067	0.99999	79.95962
	B	0.01087	0.99987	67.88447	0.00067	0.99999	79.94995	0.00067	0.99999	79.95172
Shifting	RGB	0.02947	0.99963	63.47670	0.00186	0.99998	75.45775	1.22619	0.99599	47.49072
	R	0.00938	0.99990	68.47958	0.00067	0.99999	79.99101	0.00067	0.99999	79.99281
	G	0.00938	0.99990	68.45328	0.00067	0.99999	79.98969	0.00067	0.99999	79.99153
	B	0.01145	0.99987	67.56043	0.00067	0.99999	79.96966	0.00067	0.99999	79.97147
Rotating	RGB	0.02576	0.99967	64.51397	0.00186	0.99998	75.44416	1.26586	0.99589	47.37580
	R	0.00941	0.99990	68.40671	0.00066	0.99999	80.01290	0.00066	0.99999	80.01458
	G	0.00897	0.99990	68.74466	0.00068	0.99999	79.93665	0.00068	0.99999	79.93841
	B	0.01085	0.99987	67.85772	0.00067	0.99999	79.99183	0.00067	0.99999	79.99361
Shifting-Rotating	RGB	0.02955	0.99963	63.46560	0.00187	0.99998	75.42310	1.26548	0.99602	47.33947
	R	0.00937	0.99990	68.54324	0.00067	0.99999	79.96979	0.00067	0.99999	79.97154
	G	0.00937	0.99990	68.45396	0.00066	0.99999	79.99169	0.00067	0.99999	79.99355
	B	0.01087	0.99987	68.14839	0.00068	0.99999	79.93932	0.00068	0.99999	79.94099

**Table 6:** Quality metrics results for the second secret data.

**Block structure**	**Data hidden colour channel**	**The sequential data embedding**			**The sequential data embedding in all frames**			**The non-sequential data embedding in all frames**		
		**MSE**	**SSIM**	**PSNR**	**MSE**	**SSIM**	**PSNR**	**MSE**	**SSIM**	**PSNR**
Fixed	RGB	0.02759	0.99970	63.77663	0.00509	0.99996	71.06265	0.37861	0.98924	42.57789
	R	0.00946	0.99993	68.39029	0.00175	0.99999	75.72659	0.00175	0.99999	75.72540
	G	0.00926	0.99991	68.48426	0.00177	0.99999	75.66169	0.00177	0.99999	75.66232
	B	0.01090	0.99910	67.80613	0.00175	0.99999	75.72357	0.00175	0.99999	75.72418
Shifting	RGB	0.02755	0.99970	63.92569	0.00507	0.99996	71.08231	3.64159	0.99015	42.71281
	R	0.00964	0.99993	68.30839	0.00176	0.99999	75.70106	0.00176	0.99999	75.70169
	G	0.00903	0.99994	68.61257	0.00176	0.99999	75.68123	0.00177	0.99999	75.68187
	B	0.01112	0.99991	67.69222	0.00175	0.99999	75.71196	0.00175	0.99999	75.71259
Rotating	RGB	0.02756	0.99970	63.76685	0.00509	0.99996	71.06422	3.73818	0.98921	42.68985
	R	0.00947	0.99993	68.37761	0.00175	0.99999	75.70491	0.00175	0.99999	75.70557
	G	0.00891	0.99995	68.65292	0.00176	0.99999	75.70777	0.00176	0.99999	75.70842
	B	0.01091	0.99991	67.76976	0.00175	0.99999	75.71372	0.00175	0.99999	75.71435
Shifting-Rotating	RGB	0.02749	0.99970	64.48933	0.00510	0.99996	71.05740	3.68430	0.99005	42.66160
	R	0.00962	0.99993	68.31600	0.00174	0.99999	75.72473	0.00175	0.99999	75.72723
	G	0.00903	0.99994	68.61717	0.00177	0.99999	75.68374	0.00176	0.99999	75.68440
	B	0.01111	0.99991	67.69662	0.00175	0.99999	75.71199	0.00175	0.99999	75.71262

Average and best PSNRs, MSEs, SSIMs and payloads obtained for the 1st and 2nd secret messages have been presented in Table 7.

**Table 7:** Average and the best PSNRs, MSEs, SSIMs and Payloads.

		**PSNR (dB)**	**Payload (%)**	**MSE**	**SSIM**
1st secret message	Average value	72.63878	8.2%	0.11055	0.99960
	Best value	80.01458	7.5%	0.00066	0.99999
2nd secret message	Average value	69.71885	20.3%	0.24445	0.99907
	Best value	75.72473	19.7%	0.00174	0.99999

(i) As can be seen from Table 5, for the 1st secret message, the best results were obtained from the data embedding processes based on the non-sequential secret data embedding in ‘all frames algorithm’, the rotating KBM, and the R colour channel.
(ii) As can be seen from Table 6, for the 2nd secret message, the best results were obtained from the data embedding processes based on the non-sequential secret data embedding in all frames algorithm, the shifting-rotating KBM, and the R colour channel.
(iii) As can be seen from Table 7, the average and the best imperceptibility values were obtained for the 1st secret message whereas average and the best payload values were obtained for the 2nd message.

## Discussion

It is observed from Tables 5, 6 and 7 that the MSE and PSNR decrease, and the disturbance rate increases in the stego object as the amount of the secret data or payload increases. The lowest MSE and PSNR values for each application were observed with the data embedding process performed with the RGB colour channels. It should be noted here that SSIM values for the process of embedding the data into all channels of RGB are high whatever the selections of other parameters. Considering the structural similarity rates, a high SSIM value shows that there is no perceivable difference in the image quality of the application even for all RGB channels are selected for data embedding.

It is observed that the MSE&PSNR values are high in the data-hiding processes performed using a single-colour channel of each pixel. In this case, the payload that is inversely proportional with PSNR and MSE drops as seen in Table 7.

When the MSE and PSNR quality metric values were obtained by the sequential data embedding algorithm, it was observed that there were greater degrees of change in the pixel values constituting the video frame compared to the other secret data embedding algorithms as the data were concentrated within a certain video frame. MSE and PSNR quality metric values obtained by the sequential data embedding in all frames and the non-sequential secret data embedding in all frames algorithms showed that no significant changes occurred in the video frames since the data was embedded in all frames and therefore the amount of data to be hidden within a single video frame was low as seen in Tables 5 and 6. The MSE and PSNR values are inversely proportional to the size of the data to be hidden or payload. This situation directly affects the pixel change rates in the video frames as seen in Table 7.

Regarding the SSIM values, there is no detectable difference in the image quality of the application. According to the assessment results obtained in all applications, the suggested algorithms do not cause a great disturbance in image quality. The fact that the SSIM value is very close to 1 indicates that the structural similarity between the frames is very high and that the frames are almost identical as can be seen from Tables 5 and 6.

It is observed that the best result values in the applications are obtained from the data embedding algorithms using all the video frames. Because these algorithms use all frames in data embedding, fewer changes are made in the frames. Assessment results of these algorithms showed a significant similarity between the original frame and the data-hidden frame. This situation makes it harder to notice that data is hidden within the stego object as can be seen from Tables 5 and 6.

For the first secret data, the data embedding process performed using non-sequential secret data embedding in all frames algorithm, the rotating KBM structure and, the R colour channel resulted in the most successful values. PSNR, SSIM and MSE were obtained as 80.01458 dB, 0.99999 and 0.00066. There is a 0.05% difference in PSNR between the rotating KBM (80.01458 dB) and the shifting-rotating KBM (79,97154 dB) for the R colour channel and the non-sequential data embedding in all frames for the first secret message as can be seen from Table 5. When it comes to second secret data, the data embedding process performed using non-sequential secret data embedding in all frames algorithm, the shifting-rotating KBM structure and, the R colour channel resulted in the most successful values. PSNR, SSIM and MSE were obtained as 75.72723 dB, 0.99999 and 0.00175 as can be seen from Table 6. In this context, among the methods proposed, the data embedding process performed using non-sequential data embedding in all frames algorithm, the shifting-rotating KBM structure and, the R colour channel is suggested by the author of this paper since shifting-rotating KBM structure increases data security. Additionally, the non-sequential data embedding in all frames algorithm has the possibility to embed sequential characters within irrelated frames. Thus, this property will add extra security for the secret data.

To ensure that the hidden data is affected by the negative factors that may cause a data container on the cover video, with the least loss, an equal amount of data is hidden in each video frame regarding the sequential data embedding in all frames and the non-sequential data embedding in all frames algorithms. Thus, data loss will be less in operations such as clipping and cutting that may occur in the cover video. This situation allows data losses that may occur because of frame damage (possibly encountered in the cover video) to be easily obtained from the entire text with natural language processing.

It should be noted here that depending on the cover video and secret message size other combinations might have better results. For this study, the same video that was dynamically recorded was used for all combinations. Having a good result in the R channel depending on the luminance at the moments when the video was recorded. Depending on the luminance change, embedding the secret message in any of R, G, B channels or all channels of RGB can produce better results. The authors of this paper recommend the reader to try all combinations for the secret message to discover the best results if a powerful computer is used for implementation. But, when the resistance against attacks and imperceptibility are the main concerns, the reader is advised to use shifting-rotating KBM- due to security- and to use only one channel of RGB and non-sequential secret data embedding due to imperceptibility. In this case, the payload will be dropped to an acceptable level since all the frames will be used for data hiding.

## Conclusion

For any video steganography, the good trade-off among robustness, imperceptibility and payload must be created and maintained. But, there is no unique set of properties that are satisfied by video steganography.

(i) When the combination of the non-sequential data embedding in all frames algorithm, one of RGB channels and the shifting-rotating KBM are used robustness and imperceptibility increase. Additionally, the acceptable payload is reached and the time to implement the proposed combination increases.
(ii) When the combination of the sequential data embedding, all channels of RGB and fixed KBM are used the security, robustness and imperceptibility values decrease. Additionally, payload increases and time to implement the proposed combination decreases.

Finally, the other combinations will result in between these two combinations. Thus, the proposed approach allows options to trade-off among robustness, imperceptibility and payload. But, in any case, security is higher in the proposed steganography method due to the rotating-shifting structure of KBM and logical operations when this study is compared with classical LSB and studies conducted with LSB-based approaches in the literature. This is achieved with successful imperceptibility, acceptable payload and contribution to robustness. As mentioned previously the average PSNR and payload values of the proposed method were calculated as 71.13878 dB and 14.25%. These values prove the success of the proposed method regarding imperceptibility and payload since they are still among the highest ones in the literature as can be seen from Table 1. However, the complexity of the proposed method is significant. This makes the computational time lengthy as can be seen from Table 3. Powerful graphic cards or supercomputers can be used to decrease the time taken for the embedding process especially in the services where time constraint is required.

Although the resistance of the proposed system against visual and transformation based attacks as part of the evaluation of the robustness has been demonstrated the authors of this paper are studying statistical attacks as part of the steganalysis. The most appropriate statistical algorithms such as Gaussian spread spectrum, probabilistic weighted average, simple linear averaging, salt&pepper, median filtering, block-based reconstruction *etc.* that can be applied to attack the proposed system are investigated. The results will be published as different studies since steganalysis is a different discipline.

The authors of this paper believe that KBM adds a significant level of security no matter which domain of video steganography is used. Thus, the application of KBM in the transform domain of raw video steganography will also be studied by the authors of this paper to see how the KBM will affect the security, robustness, imperceptibility and payload in the related domain.

## Supplemental Information

10.7717/peerj-cs.843/supp-1 Supplemental Information 1 Computer Code

10.7717/peerj-cs.843/supp-2 Supplemental Information 2 Message

## Additional Information and dDeclarations

### Data Availability

The following information was supplied regarding data availability: The record video data is available on figshare: HACIMURTAZAOĞLU, Murat (2021): RecordVideo.zip. figshare. Media. https://doi.org/10.6084/m9.figshare.17267852.v1
The stego video data is available on figshare: HACIMURTAZAOĞLU, Murat (2021): StegoVideo.zip. figshare. Software. https://doi.org/10.6084/m9.figshare.15138831.v1
The hiding and get message data and the computer codes are available in the Supplemental Files.
