# Supplementary figures and images for: LSB-based pre-embedding video steganography with rotating & shifting poly-pattern block matrix
Source: PeerJ Comput Sci. 2022 Jan 6;8:e843. doi: 10.7717/peerj-cs.843 (PMC8771781; doi:10.7717/peerj-cs.843)

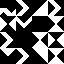

Supplement: Supplemental Information 1 [file peerj-cs-08-843-s001.zip › created KBM.png]
